# On causal roles and selected effects: our genome is mostly junk

**DOI:** 10.1186/s12915-017-0460-9

**Published:** 2017-12-05

**Authors:** W. Ford Doolittle, Tyler D. P. Brunet

**Affiliations:** 1 Department of Biochemistry and Molecular Biology, Dalhousie University, Halifax, Nova Scotia Canada; 2 Department of History and Philosophy of Science, University of Cambridge, Cambridge, UK

## Abstract

The idea that much of our genome is irrelevant to fitness—is not the product of positive natural selection at the organismal level—remains viable. Claims to the contrary, and specifically that the notion of “junk DNA” should be abandoned, are based on conflating meanings of the word “function”. Recent estimates suggest that perhaps 90% of our DNA, though biochemically active, does not contribute to fitness in any sequence-dependent way, and possibly in no way at all. Comparisons to vertebrates with much larger and smaller genomes (the lungfish and the pufferfish) strongly align with such a conclusion, as they have done for the last half-century.

## “Junk DNA” and the lungfish

The mammalian genome (haploid chromosome complement) contains roughly 3.0X10^-9^ mg of DNA, which represents about 3.0X10^9^ base pairs. This is about 750 times the genome size of *E. coli*. If we take the simplistic assumption that the number of genes contained is proportional to the genome size, we would have to conclude that 3 million or so genes are contained in our genome. The falseness of such an assumption becomes clear when we realize that the genome of the lowly lungfish and salamanders can be 36 times greater than our own …

Susumu Ohno 1972 [1]

The conundrum that Susumu Ohno, often credited as having first formally promoted the term “junk DNA”, highlighted in his 1972 paper [1] is still very much with us. We may indeed be more complex than *Escherichia coli*, but what about that lowly lungfish? Either (1) this humble creature is actually that much more complicated than a human, (2) 35/36^th^ of its DNA is unnecessary for fitness—is indeed what Ohno would have called junk—or (3) functionality is much more diffusely encoded in its giant genome than in ours, making this fish a poster-child of genotypic inefficiency. Ohno’s concept of gene was no doubt restricted compared to today’s nuanced understandings of genotype–phenotype relationships (e.g. [2]), but loosening definitions does not solve the comparison problem: why does the lungfish need so much more DNA than we do to support a phenotype that is surely no more complex than our own? And if lungfishes don’t really need so much DNA—if an equally complex and viable creature could be built with 1/36 ^th^ as much—how can we be confident that our own genomes aren’t also pretty junky? After all, *Takifugu* (formerly *Fugu*) *rubripes*, the Japanese pufferfish, gets by with one-eighth as much DNA as *Homo sapiens*, and has roughly the same number of protein-coding genes [3]. Does it really take all that additional DNA just to regulate the expression of ours?

For most of the 50 years since Ohno’s article, many of us accepted that most of our genome is “junk”, by which we would loosely have meant DNA that is neither protein-coding nor involved in regulating the expression of DNA that is. Junk was not “informational” in the sense that molecular biologists conceived that term. Such a reading was part and parcel of an understanding of the role of DNA in heredity and evolution—as the “blueprint” for cells and organisms—popular through much of the last century.

Over the nearly five decades since Ohno wrote, three general sorts of interpretations that might alleviate the pejorative connotation of “junk” but still deny any traditionally informational role for excess DNA were entertained by comparative genomicists, though not necessarily by those focused on single model organisms or humans. First, non-informational bulk (mass) roles for DNA that are relatively independent of sequence might be attributed to much non-coding and not-clearly-regulatory DNA (perhaps 90–95% of our own genomes). Graur et al. [4] would include this in the class of “indifferent” (as opposed to “literal”) DNA. Rather like ballast or “clean fill”, its function might be simply to be present (and relatively non-toxic) in a certain amount. Early on, Tom Cavalier-Smith had noted a strong correlation between DNA content and important parameters of cell biology expected to be under selection, especially nuclear and cell volume, calling this bulk role “nucleoskeletal” [5]. Such observations have been greatly extended by T. Ryan Gregory, who in 2001 suggested a “nucleotypic” role [6]. He infers that “variation in DNA content is under direct selection via its impacts on cellular and organismal parameters”, even in complex multicellular species.

Second, it has become common knowledge that the architecture (shape and configuration) of chromosomes in the nucleus matters [7–9]. Phenotypic expression of specific genes depends on where in the three-dimensional structure of a dynamic nucleus they might find themselves, and when. Gene-less regions play roles in chromosomal structuring directly and through the intermediary of long noncoding RNAs (lncRNAs) transcribed from them [10]. Nuclear architecture affects signal transduction, mechanical responses and migration of cells, and even such specific traits as vision in nocturnal animals [11]. Architectural functions are also potentially selectable in a relatively sequence-independent manner, although how the total amount of DNA affects relevant architectural features is not understood in any systematic way. What might unite architectural and nucleotypic roles, conceptually, is that it is largely the DNA itself and not its products to which such a role is assigned. And again, we must ask why the lungfish’s nuclear architecture needs to be so (apparently) expansive. Are its chromosome structure–function relationships more complex or just more diffuse?

Third, many authors also attempt to rationalize the presence of so much DNA teleologically; that is, in terms of future utility. Such DNA is proposed to be there because it *might* be useful in future. Much of the focus here has been on transposable elements (TEs) such as SINEs and LINEs, because some of these have indeed been co- opted to form regulators of the expression of other genes, seeming to justify the presence of such elements as a class [12, 13] (but see [14]). But it cannot be that the reason for the accumulation of TE copies in the genomes of a population is that some fraction of them, many generations or even speciation events down the road, might be coopted as genes or regulatory elements. There is no selective advantage to individuals within a species for doing this, and evolution has no foresight [15]! (That there *might* be an advantage to species is discussed below.)

In any case, a better explanation for the presence and abundance of such elements is readily available at the level of genomes—that they are (or were) “selfish”, the product of intra-genomic selection for survival by differential replication [16, 17]. Something like half our own genome (and presumably much more of the lungfish’s) is made up of elements that can be so explained. Some few may well have been later recruited as regulatory elements. However, retroactive justification is not only unacceptably teleological but unnecessary for explaining the existence of the vast majority of SINEs and LINEs or endogenous retrovirus-like elements.

Of course, TEs (including those in our own genome) do carry expressed and regulated genes, but these comprise adaptations benefitting their own spread, with no necessary positive contributions to the fitness of the organisms in whose genomes they reside. It is in part failure to realize or reluctance to accept that evolution by natural selection operates independently (and sometimes oppositely) at different levels of the biological hierarchy (gene, cell, organism, species) that compels many molecular biologists to deny that much of our genomes can have an evolutionary agenda independent of, or even hostile to, our own, organism-level trajectory.

“Parasitic DNAs” [17] do have functions, but for themselves, not for us. Indeed, if any long-term evolutionary benefit *is* conferred at higher levels by the presence of excess DNA riddled with TEs, it is not to individuals within species but to species within clades that the benefit might be said to accrue [18, 19]. Any selective benefit skips a level in the hierarchy. It’s not that individuals do better (differentially reproduce) within species because they have selfishly accumulating TEs, but that species harboring selfish TE-burdened individuals do better within clades (differentially speciate or avoid extinction) because, just occasionally, a copy of a TE gives rise to a genetic innovation.

Compounding a reluctance to think about natural selection as a multilevel principle, molecular biologists and genomicists often (perhaps unconsciously) subscribe to an extreme form of adaptationism—the notion that “natural selection [is] so powerful and the constraints upon it so few that direct production of adaptation through its operation becomes the primary cause of nearly all organic form, function, and behavior” [20]. Thus, many in the genomic community and the science-conscious public were gladdened by claims, widely broadcast after the publication of a collection of reports from the ENCODE consortium in 2012 [21], that functional genomics had at last put paid to the notion of junk DNA [22–24].

In a sense, the publicity around ENCODE only amplified what had become a common sentiment in the previous decade: widespread use of phrases such as “long dismissed as useless junk”, “formerly known as junk” or “formerly dismissed as junk” has been noted by Palazzo and Gregory [25]. More recently, critiques such as those described below have prompted more careful use of language by ENCODE investigators. Kellis et al. [26] for instance recognize that there is a disconnect between the several overlapping methodologies used to assess “function” and the concepts within evolutionary biology needed to give that word its meaning, and hope that further methodological refinement will clarify matters (see also [27, 28]). They write …

Our results reinforce the principle that each approach provides complementary information and that we need to use combinations of all three to elucidate genome function in human biology and disease.

There is a new caution, but still, “junk” is less popular a descriptor than it was a few decades ago, especially in biomedical research focused on our own genome. Unless we can dismiss Ohno’s conundrum, this is a mistake.

Had ENCODE’s results been based on accumulating proof that the apparent excess DNA in our genome plays nucleotypic or purely architectural roles and could not be deleted without reducing fitness, a backlash from evolutionary biologists might have been avoided. Believers in human junk might have accepted that—though lungfishes might have junk still—the human genome is just right, a sort of “Goldilocks genome”. But in fact it was more typically informational roles that were invoked by ENCODE investigators, publicizers and many biomedical researchers. The new debate over junk was based on the belief that functional genomics had overthrown the notion of junk on its original terms—as DNA that is neither structural genes nor directly involved in controlling the expression thereof. A news article in *Science* declared the “eulogy for junk DNA”, while the editors of *Lancet* [29] enthused that …

Far from being ‘junk,’ the DNA between protein encoding genes consists of myriad elements that determine gene expression, whether by switching transcription on or off, or by regulating the degree of transcription and consequently the concentrations and function of all proteins.

## What do we mean by “function”?

The ENCODE papers’ authors and the reporters recounting the project’s successes in the popular press claimed that 80.4% of human DNA is functional [21]. Measures of function were selected to be those activities common to unambiguously functional expressed protein-coding or regulatory genes.

The first problem here is simply bad logic [30]: just because phenotypically significant genes have certain characteristics does not mean that any stretch of DNA with these characteristics is phenotypically significant, that is that its expression is under the purview of natural selection, even broadly construed. That A implies B does not entail that B must imply A. Assuming that it does is especially dangerous in a system as noisy as a cell: there will likely be accidental interactions and low-level background transcription.

The second is deeper [31, 32]: it concerns the meaning of “function”. Is the mere fact that X causes Y enough to say that Y is X’s proper *function*? As a concrete example, does the fact that certain trinucleotide repeats *function* in the development of Huntington’s disease mean that *causing disease* is *the function* of these trinucleotide repeats?

Philosophers have debated such issues for millennia and, while now fully embracing Darwinian evolution, entertain two general sorts of meaning for the word “function” [31, 32]. By the first (*selected effect, or SE*), the function(s) of trait T is that (those) of its effects E that was (were) *selected for* in previous generations. *They explain why T is there.* Selected effects might change, of course, as in the purported change in the function of feathers as insulation to aids in flight. Effects selected recently trump earlier ones as explanations. Nonetheless, any claim for an SE trait has an etiological justification, invoking a history of selection for its current effect.

**Fig. 1:**
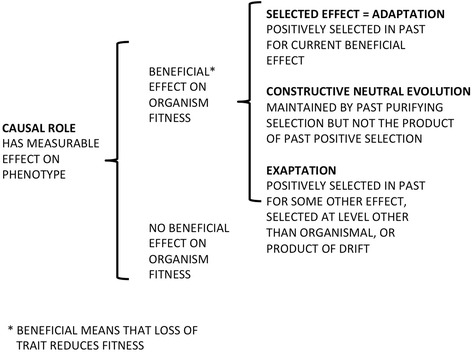
Traits attributable to DNA. We include all effects of DNA that are detectable at some level other than that of the DNA itself (its sequence and amount): that is, all expressions in biochemical, physiological or behavioral phenotype and all measures of activity employed by ENCODE. Some will have a beneficial effect on organismal fitness in that their elimination would have a detrimental effect on survival and reproduction vis-à-vis the wild type, under environmental conditions encountered by the organism. Some will not. Of the former, only selected effect functions (adaptations) were positively selected in the past for their current contribution(s) to fitness, and are now presumably under purifying selection to maintain them. The products of CNE and exaptations are similarly presumed to be under purifying selection but the former were acquired without positive selection as in Fig. 2, while the latter may be the result of positive selection for some organismal function other than their current one, or at some level other than organismal (as in the rare TEs coopted into gene regulation), or result from genetic drift

ENCODE assumed that measurable effects of various kinds—being transcribed, having putative transcription factor binding sites, exhibiting (as chromatin) DNase hypersensitivity or histone modifications, being methylated or interacting three-dimensionally with other sites—are functions *prima facie*, thus embracing the second sort of definition of function, which philosophers call *causal role* (CR; Fig. 1). As Garson [31] recently summarized it: “According to this view, roughly, a function of a part of a system consists in its contribution to some system-level effect, which effect has been picked out as especially interesting by a group of researchers.” Causal role thinking tells us what something does, not why it is there, and there is often no explicit requirement that even the “system-level” effect to which it contributes has been selected for (although Thomas [33] for one, would like to impose such a criterion). CR is not just SE in other terms: certain nucleotide repeats do play a causal role in the system that is Huntington’s disease, but they were not selected for because they do this. Oxygenated hemoglobin does make our blood red, but our ancestors did not owe their survival to the color of their blood,

The notion of “junk DNA” makes sense only in terms of SE function [32]. It assumes that much DNA lacks such a function, or at least lacks encoded “information” of the sort contemplated in molecular biology’s “central dogma”. ENCODE’s use of a much less restrictive CR definition encouraged its investigators to consider most DNA as functional. The publicity around their findings—in particular claims that the “ENCODE project writes [the] eulogy for junk DNA” [24] and the largely informational language (genes and their regulation) in which such eulogies were framed—was thus based on a conflation. A function concept embedded in one definitional framework (SE) was (supposedly) refuted by empirical data based on quite another (CR). What genomic constituents do (their activity) was assumed to be why they are there; as if natural selection was an all-seeing force and biological systems are noise- free; as if Huntington’s disease were for our own good, or blood is red because our ancestors were the more prolific depending on this coloration.

## Candidate and proper functions

It may be impossible to imagine a trait with a selected effect that does not also have that effect as a causal role, but the reverse is easy (Fig. 1). In an essay meant to clear the air in the context of a Gene Ontology framework [33], Thomas appropriately concludes that …

Thus, simply identifying a coherent, regulated system of activities can be a fruitful, practical start for identifying selected effect functions. Causal role analyses can and do play such a role in functional anatomy and molecular biology. But of course they are only candidates for evolved biological functions until they have been related to past survival and reproduction, the ultimate function of any biological program.

He calls such evolved biological functions “proper” functions, and there really is a thorny empirical issue at stake: just how much of an organism’s genome is the product of natural selection at the level of organisms (has an SE function)? Given any stretch or particular sequence of DNA we might ask …

1. Is it expressed in phenotype, this minimally defined as any detectable biochemical, developmental or behavioral effect beyond its mere presence and sequence as DNA (thus discounting such obviously trivial consequences as GAATTC being a site for the restriction endonuclease Eco R1)?
2. Does such expression *currently* make a positive contribution to organismal fitness?
3. Does past positive selection for such a contribution to organismal fitness account for the presence or sequence of the DNA?

If all answers are yes, the DNA is “functional” in the SE sense, although we still might want to distinguish DNA whose function is sequence-dependent and “informational” from DNA whose primary role is simply as bulk, or to support nuclear architecture, as discussed above. The ENCODE project addressed only question 1, and often even “functional” analyses targeting specific genetic or cellular components and involving careful documentation of the biochemical or physiological consequences of alteration or removal of the element in model organisms lack confirmation, through comparative genetic studies, of any fitness effects (question 2).

Moreover it is not at first clear what we should make of cases in which the answers to questions 1 and 2 are known to be “Yes” but to 3 is “No” or “Unknown”. That is, how do we classify traits whose elimination would likely (or demonstrably does) reduce fitness but for which we can imagine no credible and relevant historical scenario that entails such fitness loss. The near-sighted among our Pleistocene ancestors might have had more progeny if they had worn eyeglasses, but surely our noses did not evolve so that in future we’d have something to keep such visual prostheses from falling off our faces. Gould and Vrba [34] coined a term for this in 1982—“exaptation”. For them, “adaptations” are characters shaped by natural selection for their current use, while “exaptations” were shaped by natural selection for some use other than their current one, or were not shaped at all by selection and are the consequence of some other process, such as genetic drift. This formulation is fully consistent with SE definitions of function, and with treatments of “adaptation” that depend on them, for instance that of the philosopher Robert Brandon [35] who would say that exaptations exhibit “adaptedness” but not “adaptation”. He writes …

A trait may appear with one mutation. Such a trait is not an adaptation regardless of its effect on the adaptedness of its possessor. If the trait does increase the adaptedness of its possessor and thereby increases in frequency in the population it will then become an adaptation.

Not only is a past history of selection necessary for a trait to be an adaptation, it must be the right sort of history. Invasive species may be particularly well adapted to new environments that lack predators, but this beneficial consequence is not an adaptation.

This is not simply philosophical hair-splitting, if we seek to know *why* an organism or a gene or a genome has a characteristic that it does as a “proper function”. This is surely one of the ultimate motivations for a project like ENCODE, and explains much of the enthusiasm for the 80.4% functional claim. Moreover, suppose we have answered questions 1 and 2 positively and 3 negatively. Question 3 permits to several possible negative answers that nevertheless say something of interest about natural selection. The trait might derive from selection at a level lower (“selfish” TEs) or higher (species selection, as for sexual reproduction), be the product of drift in small populations, or result from constructive neutral evolution (CNE) [36]. The first three seem to be adequately accommodated by Gould and Vrba’s term “exaptation”, but the last is of ambiguous status in our classifications, was not addressed by Gould and Vrba and has not been considered by the philosophical community that gave us the distinction between causal roles and selected effects.

CNE results in the maintenance by purifying selection of traits that were never under positive selection. It recognizes the logical (and biological) fact that although some traits that arose by positive selection are now maintained by purifying selection, not all traits maintained by purifying selection arose by positive selection.

**Fig. 2:**
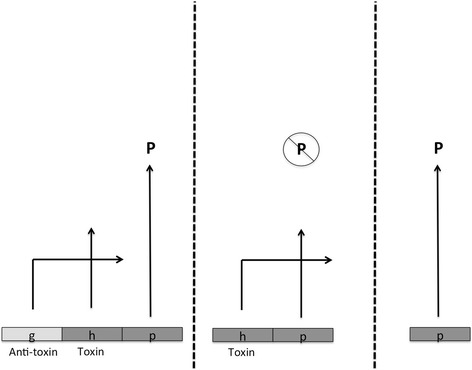
Constructive neutral evolution as illustrated by a simple toxin–antitoxin system. Shown are *g* (the antitoxin gene), *h* (the toxin gene) and *p* (a gene producing beneficial phenotype P). When both *g* and *h* products are present the phenotype P is expressed, since the *g* product suppresses (inhibits) the toxic effects of the *h* product. When *g* is deleted, *h*-product toxically inhibits expression of P, leading to the inference that *g* is essential for the function of P and may have undergone positive selection for the performance of P. But if *g* in fact were present before the *h-*product acquired its potential toxicity against P, and “pre-suppressed” such toxicity, no positive selection need be invoked. The suppression by chaperones of the effects of mutations in target proteins is an example of such CNE. In many other systems a simpler evolved dependence of one component “A” on another “B” permitted by the “pre-suppressive” activity of the second on possible, otherwise deleterious, mutations in the first can be imagined [38]. Again the conclusion that B evolved positively to support the activity of A would be unwarranted

Toxin–antitoxin pairs provide a good example. Imagine that gene *g* encodes an anti-toxin for the product of another (“toxic”) gene *h*, and the deletion of *g* allows the product of *h* to have some disruptive effect on fitness-promoting phenotype P, while the deletion of both *g* and *h* would rescue the development of P (and have no other deleterious effects). Fig. 2 illustrates such a scenario, and in cases where the toxic effect of h evolved *after* the antitoxin activity of g, no positive selection need ever have occurred. Such a scenario is not unimportant or hypothetical. For instance, the prior presence of chaperones (as the product of g, and possibly selected for to correct errors in just one or a few proteins) allows for the subsequent evolution of many intrinsically less stable variant proteins (h-gene products) whose functions then become chaperone-dependent. Moreover, protein products of h genes, if accumulated without chaperone activity, could, as aggregates, impede cell growth. In either case, deletion of the chaperone could be detrimental through its effect on many cellular constituents, but it is not the case that the chaperone first appeared to prevent this widespread detriment. Instead chaperones, once present, permit the increasing dependence of other proteins upon their activity [37].

Selection is involved in such a story, but it is purifying selection against the loss of a component or activity (the chaperone), not positive selection for its presence. Accounts of selected effect function fail to deal with such situations. It is problematic whether we should regard CNE-derived traits, a possibly very important contributor to complexity [38], as “proper functions”. Here (Fig. 1) we place such traits in a separate category. Although they resemble both selected effects (in having a history of selection, albeit purifying rather than positive, that explains why they are there) and exaptations (in being present for reasons other than positive selection for their current role), they are not clearly either.

In any case, much contemporary molecular biological research comprises experiments showing that elimination or alteration of trait X has phenotypic consequence Y, regardless of fitness effect, negative or positive. Only question 1 (“Is it expressed in phenotype, this minimally defined …”) is answered, thus identifying a CR function, though not necessarily the primary one (first in its causal chain). Sometimes question 2 (“ Does such expression currently make a positive contribution to organismal fitness?”) is also answered, or a positive answer is assumed. Many traits passing these tests may be excellent candidates for what Thomas [33] calls “proper functions” (selected effects). But what we need to decide this are not more experimental data from model systems but comparative data of the sort next described, bearing on question 3 (about past positive selection). As Ponting [28] concludes, “population genomics-based approaches to predict function are paramount because, counter-intuitively, experiments are not perfect predictors of function.”

## Sorting candidates for proper function in our own genomes

Causal role functions are easier to establish than selected effects. Once an effect of some trait (say a gene) on a system of interest (usually a cell type, tissue or whole organism) is identified—through mutagenesis, gene knockout or insertion (CRISPR) experiments, or any of the correlated activities identified by ENCODE—then the gene is identified as having a causal role (“candidate function”), sometimes a very specific one, in the system. It nevertheless remains only a candidate SE-function until the relevance of the gene–effect pair to the selective history of the system (organism) has been established, and this remains very difficult. Inter- or intra-specific sequence comparisons are essential, but may show higher, lower or no significant rate differences from neutrally drifting regions [28], depending on whether selection is positive (acquisition of new function or genetic arms races), purifying (ridding populations of deleterious mutations) or sequence-independent (spacers between binding sites, for instance). Ironically, although CR studies can tell us what a particular DNA sequence does, and sequence comparisons may reveal that it has a history of positive selection, concluding that what a sequence does is why it is there—that any particular CR function is its SE function—is not straightforward. A convincing SE story requires the consilience of experimental and comparative sequence data as well as believable scenarios about past ecological and environmental settings. Without the last, we might assume that prominent noses arose in our Pleistocene ancestors to support their eyeglasses.

One comparative approach to identifying genetic traits that are under either positive or purifying selection requires showing a non-neutral ratio of non- synonymous to synonymous amino acid substitutions [39] for exons. Others seek genomic regions that have greater sequence conservation than expected inter- or intraspecifically (given an estimate of background mutation frequency). If a region of the human genome shows more similarity to its homolog within a closely related species than would be expected from the lack of selective constraint and the background mutation rate, then presumably the region is under selection, or the actual background mutation rate is less than expected. Recent selection, affecting only the differential reproductive successes of variants within our species, requires intraspecific population studies, soon to become adequate in scope. Ponting [28] writes …

Human genome sequencing at the population level is now accelerating. The resulting extensive diversity data will permit the inference of constraint at high resolution and will thus shed light on function and molecular mechanisms. It will also help to overthrow misguided notions that function requires between-species sequence conservation *or that function is widespread outside constrained sequence (emphasis ours).*

While there is currently an atmosphere of revisionism about mutation rate and estimates of conservation, even when the suggested revisions are upward they do not begin to approach ENCODE’s 80.4% functionality. Early estimates of conservation among mammals from comparison with mouse genomes provided a value of ~ 5%, significant primarily since it is almost triple that expected to be protein-coding. For examples of revisionism, Lunter et al. [40] downwardly revised this estimate to between 2.56 and 3.25% using a model of indels and comparisons of human, mouse and dog genomes, then revised the model and estimate to 8.2% in [41]. Eöry et al. [42] on the other hand report a value of 5.4% for the portion of nucleotides under effective negative selection. A much higher estimate was offered by Pheasant and Mattick [43], obtained by accounting for (1) a supposedly two-fold upward bias introduced by unappreciated positive selection and turnover, and (2) relying on the largest of three estimates (“loose”, “moderate” and “strict”) given in Margulies et al. [44] to arrive at a figure of 20%. Even such a generous estimate is four-fold lower than ENCODE assessments, and other moderate, conservative or strict estimates are still an order of magnitude shy.

Of course, if conservation is *not* apparent between two putatively homologous regions, this does not imply the absence of function: the same function could be accomplished by different sequences or be sequence-independent but still selectively constrained, as stretches of a required length between two binding sites might be. Thus, comparative assessment of conservation as a direct assessment of SE-function is sufficient (assuming suitable estimates of mutation rate) but not necessary to establish SE-function. Conservation amongst closely related species offers only a lower bound for the extent of selection in humans. It also cannot directly tells us what the selected functions were, and finesses such questions as, “If only 10% of the sequence of a gene is conserved in sequence, but deletion of the gene is detrimental, as is the case with some sequences encoding regulatory lncRNAs [45], what fraction do we say has function?”

Purely theoretical estimates based on population genetics principles, genome size and inferred population size are possible and offer a principled alternative, given such uncertainties. And again, these fall far short of 80.4%. A theoretical population genetic approach very recently led Graur [46] to the conclusion that “the functional fraction within the human genome cannot exceed 25%, and is probably considerably lower.” Surely for the lungfish that value would be even lower still.

## It all comes back to the lowly lungfish

No discussion of the extent to which natural selection impacts the human genome can avoid coming back to the lungfish, we contend. No matter how subtly we define function in our own genome we need to consider the general question in a general (non-anthropocentric) context, one in which we are not biologically special. (Gregory argues similarly, but prefers onions to lungfish as his comparator [25].) A simple thought experiment is to ask what might an ENCODE-type assessment targeting the lungfish reveal: would its DNA also prove to be 80% “functional”? Even if we expand our understanding of what is “under selection” in genomes to include purely structural components for which only their presence matters, and include as well features that might never have been directly selected for at the organismal level but that could not now be harmlessly removed (some exaptations and the products of CNE; Fig. 1), we need to ask why the lungfish needs *so* much more DNA.

The question revives a debate that was active immediately after the publication of the “selfish DNA” papers. Is the mass of DNA in a cell, linked to developmentally optimizable parameters of cell and nuclear size and division time, under selection because these parameters are? And if so, do various DNA- adding mechanisms such as selfish proliferation of transposable elements simply rise to the occasion, providing relatively harmless “clean fill” when needed? Or instead do such lower-level evolutionary mechanisms generate an upward pressure on the amount of DNA, countered by selection at the organismal level only when the cost of carriage of so much DNA with so many troublesome selfish elements becomes too much to bear? Surely, as with many biological questions that have two opposing answers, the truth is somewhere in the middle, and given how much DNA some species exhibit, this is considerable territory. And there is a third option: that the excess DNA generated by transposition and “accidental processes” like unequal crossing over quickly becomes recruited into a looser form of something like “functionality”, as has for instance been argued for lncRNAs by Ponting et al. [45].

lncRNAs are transcribed from most of the DNA of many vertebrate genomes and some of these, through some small fractions of their sequence lengths, engage in “regulatory” interactions with other DNAs, RNAs or proteins that are likely under selection. That many of the interacting regions are poorly conserved between species does not preclude their “functionality” within species, and Ponting et al. [45] suppose that, in aggregate, lncRNAs are functional.

On the one hand, the low degree of sequence constraint and the current absence of associations to disease might be argued to imply that lncRNAs contribute little to a species’ biology. On the other hand, because large numbers of lncRNAs, when considered together, exhibit signatures of evolutionary constraint it is apparent that past mutations in functional sequence have been deleterious and have thus been preferentially purged from populations. Taken together these observations imply that each lncRNA contributes, albeit only slightly, to an organism’s fitness, yet large numbers of lncRNAs contribute substantially when they are considered in aggregate. If so, then only rarely would obvious phenotypes arise when the transcription of a single lncRNA is disrupted, and thus only rarely will the mechanisms of individual lncRNAs be determined from simple experiments.

Note here that the inferred diffuse, aggregate role is still arguably “informational” in that it is not simply the bulk of the DNA that is functional, but the transcribed RNAs themselves that are considered relevant.

Let's now extend such thinking to the lungfish. Assume—as seems reasonable—that an ENCODE-like project directed at its genome would show that a comparable fraction of DNA is transcribed. We would have to water such “aggregate functionality” down still another 36 times. And assume—as again seems reasonable—that genome expansions in the lungfish lineage involved the proliferation of TEs or other DNA not previously engaged in aggregate regulatory function. We’d have to imagine that their recruitment into such a regulatory role was immediate—if we wanted to argue that during such expansion (still ongoing for all we know) there was no junk in this genomic lineage—at least transiently.

Surely this is unreasonable, and some fraction of the roughly 50% of our genome (and an even higher proportion of larger genomes) that is TEs was once—and probably is still—irrelevant to fitness at the level of the organism. As Ponting [28] concludes …

Most non-conserved sequence lies within the non-functional ~92% of the mammalian genome. Rapid resculpting of mammalian genomes is dominated by lineage-specific insertion and deletion of transposable element (TE) sequence whose debris, together with other repetitive sequence, contribute up to two-thirds of the human genome. Although occasionally it is proposed that a large fraction of TEs are functional, there is no evolutionary or experimental evidence to support this. Conversely, because the locations of insertion or deletion mutations in TEs occur almost exactly as would be expected from random events, the vast majority of TEs appear to be inert, with less than 2% of TE sequence (approximately 20 Mb) bearing the signature of constraint.

In the end, even if we abandon the requirement for an “informational” role for functional DNA and fully accept nucleotypic and architectural roles as selected effects, it is the irrelevance of the majority of TEs (at least half of our own DNA) to fitness at the organismal level that means that “junk” is likely always to be a reasonable way to refer to it.

## What makes us human?

Moreover, fitness must be defined more carefully than being necessary for the range of phenotypes currently exhibited by the human population. Many specific differences between human and chimpanzee DNA are no doubt necessary for the phenotypic distinctions between us and them (and thus are CR-functional), but other phenotypes might be equally viable or fit. We and chimps are not the only possible apes, and many of our adaptednesses may not be adaptations at all. That is to say, many of our uniquely human traits are probably the contingent products of drift and historical accidents—they make us us, but were not selected to have that, or necessarily *any*, effect. So we are back to Ohno’s conundrum. If lungfishes have junk, or at the least extraordinarily weakly or diffusely functional DNA in their genomes, why is it that we think we do not?
